# Antioxidant Activity Evaluation of Dietary Flavonoid Hyperoside Using *Saccharomyces Cerevisiae* as a Model

**DOI:** 10.3390/molecules24040788

**Published:** 2019-02-22

**Authors:** Yuting Gao, Lianying Fang, Xiangxing Wang, Ruoni Lan, Meiyan Wang, Gang Du, Wenqiang Guan, Jianfu Liu, Margaret Brennan, Hongxing Guo, Charles Brennan, Hui Zhao

**Affiliations:** 1 Tianjin Key Laboratory of Food and Biotechnology, School of Biotechnology and Food Science, Tianjin University of Commerce, Tianjin 300134, China; gyt19920908@126.com (Y.G.); flianying@126.com (L.F.); wangxiangxing@kj-lab.cn (X.W.); 13820344450@163.com (R.L.); wangmeiyan@tjcu.edu.cn (M.W.); dugang@tjcu.edu.cn (G.D.); guanwenqiang@tjcu.edu.cn (W.G.); ljf@tjcu.edu.cn (J.L.); 2 Centre for Food Research and Innovation, Department of Wine, Food and Molecular Bioscience, Lincoln University, Lincoln 7647, New Zealand; Margaret.Brennan@lincoln.ac.nz; 3 The Third Central Clinical College, Tianjin Medical University, Jintang Road, Hedong, Tianjin 300170, China; kuohx@hotmail.com

**Keywords:** hyperoside, oxidative damage, lipid peroxidation, intracellular ROS, *Saccharomyces cerevisiae*

## Abstract

Oxidative stress leads to various diseases, including diabetes, cardiovascular diseases, neurodegenerative diseases, and even cancer. The dietary flavonol glycoside, hyperoside (quercetin-3-*O*-galactoside), exerts health benefits by preventing oxidative damage. To further understand its antioxidative defence mechanisms, we systemically investigated the regulation of hyperoside on oxidative damage induced by hydrogen peroxide, carbon tetrachloride, and cadmium in *Saccharomyces cerevisiae*. Hyperoside significantly increased cell viability, decreased lipid peroxidation, and lowered intracellular reactive oxygen species (ROS) levels in the wild-type strain (WT) and mutants *gtt1∆* and *gtt2∆*. However, the strain with *ctt1∆* showed variable cell viability and intracellular ROS-scavenging ability in response to the hyperoside treatment upon the stimulation of H_2_O_2_ and CCl_4_. In addition, hyperoside did not confer viability tolerance or intercellular ROS in CdSO_4_-induced stress to strains of *sod1∆* and *gsh1∆.* The results suggest that the antioxidative reactions of hyperoside in *S. cerevisiae* depend on the intercellular ROS detoxification system.

## 1. Introduction

Oxidative stress reflects an imbalance between an organism’s excessive production of oxygen radicals and its reduced capacity to detoxify. Oxidative intermediates, such as OH**•**, O**•**^2**−**^, and NO**•**, attack cell components and cause inflammation, cancer, ageing, and metabolic diseases [1,2]. It has been commonly accepted that the prevention of oxidative stress benefits human health [3,4]. For example, the ingestion of natural plants such as vegetables, fruits, and tea [5,6,7,8,9,10] has been well identified to counteract the amount of oxidative stress that cells encounter and to reduce the incidence of diseases related to oxidative damage [11,12,13,14]. The positive effect of a phytogenic diet on human health can be mainly attributed to the abundance of bioactive flavonols with pharmaceutical properties.

**Figure 1:**
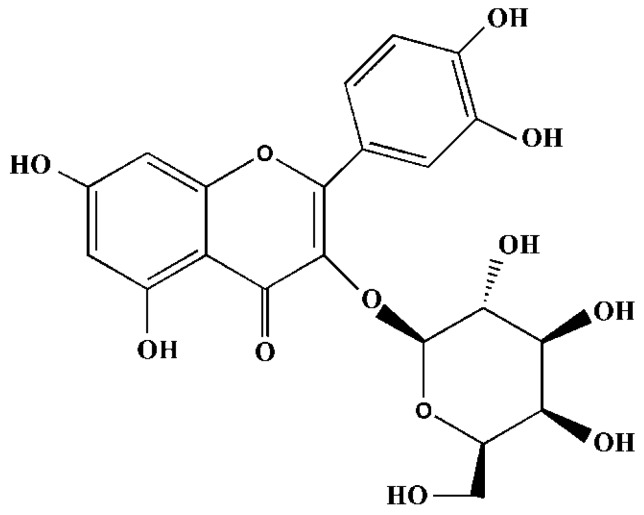
The structure of hyperoside.

Within the flavonoid group, hyperoside (quercetin-3-*O*-β-d-galactopyranoside, Figure 1) is a member of the family of *Hypericaceae*, *Rosaceae*, and *Labiata* and the genus of *Crataegus* [15,16,17]. Hyperoside has been suggested to attenuate oxidative stress-related diseases [18,19]. For example, Piao elucidated the cytoprotective effects of hyperoside against H_2_O_2_-induced cell damage in Chinese hamster lung fibroblast cells, in which the scavenging mechanisms include elevation of the expression of HO-1 and activation of nuclear factor erythroid 2-related factor 2 (Nrf2). Furthermore, Xing [13] suggested that hyperoside prevents oxidative damage through GSK-3β inactivation. Of note, the major site of generation of reactive oxygen species (ROS) is located in the mitochondrial respiratory chain (RC), where the surrounding molecules (i.e., superoxide dismutase (SOD) and cytosolic catalase 1 (CTT1)) react with the electrons of free radicals in regulating the cellular bioenergetic status under physiological or pathological conditions. Mitochondrion usually acts as the target responding to oxidative stress, resulting in the functional decline of cells. It has been suggested that hyperoside regulates the mitochondrial apoptotic pathway to prevent oxidative damage [20,21]. However, few studies have investigated the role of hyperoside in mitochondrial detoxification for the prevention of oxidative stress.

In the present study, we used *Saccharomyces cerevisiae* as a model organism for studying antioxidant protection because yeast has developed antioxidant defence systems, including an enzymatic ROS detoxification system that involves SOD, CTT1, GPX, and a non-enzymatic system mainly including glutathione (GSH), to maintain intracellular redox status and ROS levels [2]. Therefore, we attempted to validate the response mechanism by which hyperoside decreases oxidative damage in *S. cerevisiae* (BY4741WT and its isogenic mutants *gsh1∆*, *ctt1∆*, *sod1∆*, *gtt1∆*, *gtt2∆*) caused by oxidants (CdSO_4_, H_2_O_2_ and CCl_4_), and to provide further evidence for hyperoside to be used as a health food product.

## 2. Results and Discussion

### 2.1. Cytotoxicity Assay of Hyperoside

**Figure 2:**
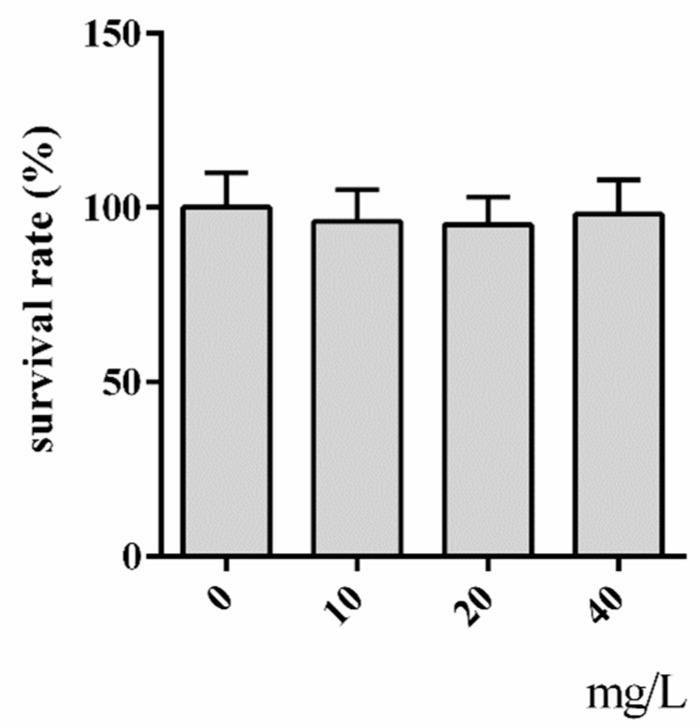
Survival of exponential WT cells exposed to increased concentrations of hyperoside.

To evaluate the toxic impact of hyperoside on the yeast cells, we first determined the cell viability of WT cells exposed to hyperoside for 2 h. As expected, we did not find hyperoside to be toxic to the yeast cells within the observed range of 10–40 mg/L, and the cells continued to reach 100% tolerance (Figure 2).

### 2.2. Hyperoside Increases Oxidative Stress Tolerance in S. cerevisiae

**Figure 3:**
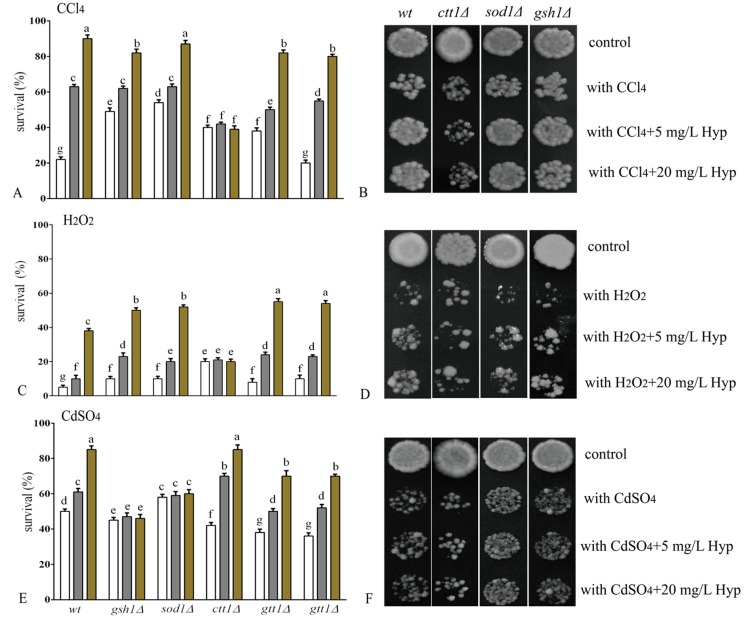
Effect of hyperoside on yeast cells under indicated stress. **A**, **C**, **E**: Effect of 10 mM CCl_4_, 2 mM H_2_O_2_, and 3 mM CdSO_4_ on the viability in *Saccharomyces cerevisiae* cells (wild type and mutant strains *ctt1Δ*, *sod1Δ*, *gtt1Δ*, *gtt2Δ*, and *gsh1Δ*) and the anti-oxidative effect of hyperoside pre-treatment. The concentration of hyperoside used was 0 mg/L (white bars), 5 mg/L (grey bars), and 20 mg/L (brown bars). **B**, **D**, **F**: Effect of 10 mM CCl_4_, 2 mM H_2_O_2_, and 3 mM CdSO_4_ on the growth of *S. cerevisiae* cells (wild type and mutant strains *ctt1Δ*, *sod1Δ*, and *gsh1Δ*) and the anti-oxidative effect of hyperoside pre-treatment. The results represent the means ± SD from three independent experiments. The statistically different results were represented by different letters in each oxidative stress group, *p* < 0.05.

The stimuli used in our study are well-recognized oxidants of cells [22,23,24]. The survival rate of the WT strain consistently dropped significantly when exposed to the oxidants CCl_4_, H_2_O_2,_ or CdSO_4_ (Figure 3). However, when the cells of the WT strain were preadapted with hyperoside, there was a concentration-dependent tolerance to all stresses observed in our experiments (Figure 3). The data suggested a protective effect of hyperoside in yeast against oxidative stimulation.

In theory, yeast cells control the intracellular redox balance through enzymatic and non-enzymatic defence systems [2]. To determine the defensive targets of hyperoside in yeast, a range of isogenic mutant strains (deficient in the genes CTT1, SOD1, GSH, GTT1, or GTT2), which present the phenotypes of enzymes involved in ROS detoxification, were used to assess the responses to oxidative stimuli.

Initially, the effect of hyperoside on the survival inhibition of CCl_4_ in the yeast strains was determined. The toxicity of CCl_4_ is usually caused by its reductive dehalogenation by cytochrome P450 into trichloromethyl free radicals (·CCl3) and peroxy trichloromethyl radicals (OOCCl3). Trichloromethyl free radicals react with sulfhydryl groups (glutathione and proteinthiols) and antioxidant enzymes (CAT and SOD), resulting in the excessive generation of ROS and membrane lipid peroxidation [22]. When hyperoside was used in combination with cells exposed to CCl_4_, an increased tolerance was observed in the strains of *gsh1∆*, *sod1∆*, *gtt1∆* and *gtt2∆* with improved survival rates of 30~60%. The mutant strain *ctt1∆* showed a much weaker response to the addition of hyperoside compared with the other strains mentioned above (Figure 3A); no difference in growth was observed for *ctt1∆*, as shown in Figure 3B. Thus, the data indicated that hyperoside-induced prevention of CCl_4_ may involve intercellular catalase.

Intercellular H_2_O_2_ or its derivatives OH• and O•^2−^ are tightly linked and attack cell membrane components, proteins, and DNA [25]. Given the importance of H_2_O_2_ in cell damage and death, we determined whether hyperoside was capable of increasing the tolerance of yeast trains to H_2_O_2_. Hyperoside improved the viability of the strains *gsh1∆*, *gtt2∆*, *sod1∆*, and *gtt1∆*, whereas this action was not observed in the mutant strain *ctt1∆* (Figure 3C); the same condition can be observed in Figure 3D. The data suggested that the protective effect of hyperoside against H_2_O_2_-induced stress may be related to catalase, which catalyses H_2_O_2_ to generate H_2_O and O_2_ to alleviate tissue injury [26].

The heavy metal cadmium (Cd^2+^) is extensively found in food materials due to its industrial use. Environmental and occupational exposure to Cd^2+^ may be an important risk factor for a broad spectrum of illnesses affecting body organs such as the liver, kidneys, and cardiovascular systems [27]. Growing evidence suggests that oxidative stress induced by Cd^2+^ alters the free radical-scavenging effects of *N*-acetyl cysteine, resulting in cellular damage via increasing genome mutations and recombination, and accelerating lipid peroxidation [23]. Consistent with the findings of CCl_4_ and H_2_O_2_, cadmium (Cd^2+^) also significantly inhibited the growth of yeast cells, as shown in Figure 3E. Treatment with hyperoside significantly improved the viability of both the wild type and mutant strains, including *ctt1∆*, *gtt1∆*, and *gtt2∆*. However, the *gsh1∆* and *sod1∆* strains did not improve sufficiently in response to hyperoside, indicating that the protective effect of hyperoside against CdSO_4_-induced stress was related to glutathione (GSH) and superoxide dismutase (SOD1) (Figure 3E,F). Interestingly, the results suggest potential solutions to cadmium toxicity, which occurs in the body mainly through decreasing cellular SOD activity and GSH expression [28].

### 2.3. Hyperoside Attenuates the Level of Intracellular Oxidation

Intercellular ROS are usually generated from the mitochondria, and excessive accumulation of ROS is characterized by the presence of highly reactive molecules that regulate cell death and ageing [29]. Previous studies have demonstrated that the ability to scavenge intercellular ROS from natural compounds plays an important role in mediating the viability of organisms in response to oxidative stress [30,31,32]. Hence, the mechanism whereby hyperoside protects against oxidative stress-induced loss of tolerance in yeast cells may be mediated by the clearance of intercellular ROS.

Our results confirm that hyperoside increased oxidative stress tolerance in *S. cerevisiae*. In particular, the intercellular antioxidant enzyme CTT1 was important for modulating oxidative stress tolerance in response to CCl_4_ and H_2_O_2_, whereas the other two enzymes, SOD1 and GSH, were responsible for cell tolerance in response to CdSO_4_. Therefore, we checked whether the effects of intercellular ROS and antioxidant enzymes were consistent with the above performances of the yeast phenotypes. The fluorescence intensity of the H_2_DCF-DA probe was used to indicate the intracellular ROS level and significantly increased when the cells were treated with the indicated oxidants (Table 1). The addition of hyperoside reversed the increase in intracellular ROS intensity in all the strains we tested. Furthermore, CTT1 deficiency neutralized the influence of hyperoside on intercellular ROS in response to CCl_4_ or H_2_O_2_ stimulation. The expression of cytosolic catalase CTT1 is thus usually required for detoxification in *S. cerevisiae* upon exposure to a variety of stress conditions, and reduces intracellular free radicals [33,34].

In fact, the toxicity of CCl_4_ and H_2_O_2_ is largely attributed to their ability to morph deftly into ROS. Via the reductive dehalogenation reaction, CCl_4_ can be transformed into trichloromethyl free radicals by cytochrome P450, which readily interacts with molecular oxygen and creates trichloromethyl peroxyl radicals [35]. With regard to H_2_O_2_, it can produce hydroxyl radicals, the most reactive and toxic ROS. CTT generally stays at a very low level in cells, but can be dramatically activated by peroxides over the threshold concentration, thereby maintaining a controlled intracellular peroxide concentration and protecting membranes from oxidative damage [36]. Thus, intercellular CTT1 deficiency might remain at a high ROS level in yeasts, even with hyperoside pre-adaption.

In contrast to CCl_4_ or H_2_O_2_ stimulation, SOD1 or GSH deficiency significantly counteracted the reduction of intercellular ROS by hyperoside, resulting in a response to CdSO_4_ stimulation, which is in agreement with the results shown in Figure 3E,F. Although Cd^2+^ is not a redox-active metal, it mediates in ROS production indirectly by weakening the enzymatic (e.g., SOD, glutathione peroxidase (GPx), and glutathione reductase (GR)) and non-enzymatic (e.g., small molecule antioxidants: reduced glutathione (GSH), vitamins C and E) antioxidative barrier of cells [37,38,39,40]. Indeed, the antioxidant enzyme SOD1 is located in the cytoplasm and mitochondrial membrane space *sod1∆* during abnormal oxidative damage because amino acids containing met and lys are hyperoside-sensitive to oxidative stress [41]. Furthermore, as the most abundant sulfhydryl donor, γ-glutamylcysteinylglycine (GSH) is an essential metabolite in both rodent and yeast cells that acts as a cofactor for the antioxidative enzymes glutathione peroxidase and glutathione transferase [42]. In the absence of exogenous GSH, cells with mutants lacking GSH are deficient in resisting toxic attacks caused by heavy metals such as cadmium [43].

Except hyperoside, a wide spectrum of polyphenols demonstrated antioxidant activity to fight against toxicities from heavy metals including cadmium [37,44,45,46,47]. The antioxidative and chelating properties were presented to explain the protective action of polyphenolic compounds against Cd^2+^ toxicity [48]. On the one hand, polyphenols directly increased the activities of antioxidative and pro-oxidative enzymes. On the other hand, polyphenols had the capacity to chelate Cd^2+^ and lower the cell burden of Cd^2+^. Our current study suggests that hyperoside exerts anti-oxidative protection on yeasts. However, whether or not the detailed action mechanisms of hyperoside against Cd^2+^ are mediated via the above pathways still needs further investigation.

### 2.4. Hyperoside Attenuates *S. cerevisiae* Cell Membrane Lipid Peroxidation

In addition to the above intercellular oxidative enzymes, cellular membrane lipids, mainly unsaturated fatty acids, represent common targets of oxidative attack. Free radicals resulting from oxidative stress, such as CCl_4_, H_2_O_2_, and CdSO_4_ [49,50,51], initiate a cascading lipid peroxidation process and eventually lead to membrane damage, including leakage and even cell death [52,53]. Because hyperoside possesses the flavonoid chemical structure of a potential hydrogen donor, we inferred that hyperoside may serve as a protector of membrane fatty acids against lipid peroxidation [54].

To further check the speculation regarding the protective role of hyperoside, membrane lipid peroxidation in yeast cells subjected to CCl_4_, H_2_O_2_, or CdSO_4_ was examined. As expected (Table 2) for the WT strains, the membrane lipid peroxidation level of the yeast cells was significantly increased under the stimulation of the three oxidants. The WT yeast cells were also re-evaluated with hyperoside prior to culturing with the oxidant, and alleviation of oxidative damage to membrane peroxidation was observed, which was in accordance with the tolerance assay (Figure 3) and indicated the capacities of hyperoside for increasing the integrity of the cell membrane.

Having identified the attenuation of lipid peroxidation in the WT yeasts, we investigated possible cellular enzymes involved in the redox regulation of membrane lipids. Hyperoside was observed to protect the lipid peroxidation in yeast cells from oxidative stress regardless of the antioxidant enzyme (Table 2). Indeed, it is suggested that the antioxidant enzymes located in the cytoplasm or mitochondria lack definitive preventive effects on membrane lipids [2], and the potential mechanism is worth exploring further.

Interestingly, lipid peroxidation caused by Cd^2+^ was more alleviated in the mutants, including *sod1∆*, *ctt1∆*, and *gsh1∆* than in the WT strain. This could be attributed, at least partly, to the super expression of other antioxidant systems as a form of compensation. Several other studies indicated that a deficiency in one antioxidant system may be overcome by an increase in the remaining defence system [55,56].

## 3. Materials and Methods

### 3.1. Chemicals and Reagents

H_2_O_2_, CdSO_4_, and CCl_4_ were purchased from YuanLi Chemical Co., Ltd. (Tianjin, China). Dimethyl sulfoxide (DMSO), yeast extract, and peptone were purchased from Oxoid Company (Basingstoke, UK). Glucose was purchased from Amresco (Solon, OH, USA). Hyperoside was purchased from Biopurify Technology Development Co., Ltd. (Chendu, China), the structure and the purification (99.13%) of hyperoside was confirmed by ^1^H-NMR and ^13^C-NMR and MS (Figures S1–S4. Tables S1 and S2).

### 3.2. S. cerevisiae Strains, Media, and Growth Conditions

The wild-type (WT) strain of *S. cerevisiae* BY4741 (Matα his3∆_1_ leu2∆_0_ met15∆_0_ ura3∆_0_) [57] was purchased from EUROSCARF (European S. cerevisiae Archive for Functional Analysis, Institute of Molecular Biosciences, Johann Wolfgang Goethe-University Frankfurt, Frankfurt, Germany), and its isogenic mutants *ctt1∆*, *sod1∆*, *gsh1∆*, *gtt1∆*, and *gtt2∆* harbouring the genes CTT1, SOD1, GSH1, GTT1, and GTT2, respectively, were obtained from Dr. Pereira (Departamento de Bioquímica, Universidade Federal do Rio de Janeiro, Rio de Janeiro, Brazil). Stocks of the strains were maintained on solid Yeast Extract-Peptone-Dextrose (YPD) (1% yeast extract, 2% glucose, 2% peptone, and 2% agar), and the medium also contained 0.02% geneticin for the mutant strains. For all experiments, the cells were grown in liquid YPD medium using an orbital shaker (Aosheng, Hangzhou, Zhejiang, China) at 28 °C/160 rpm.

### 3.3. Cytotoxicity Assay of Hyperoside

Yeast cells in the mid-log phase (10^6^ cells/mL) were reinoculated in fresh medium (the initial cell concentration was 10^5^ cells/mL) with or without hyperoside (10 mg/L, 20 mg/L, or 40 mg/L) and incubated at 28 °C/160 rpm for 2 h. Then, diluted (1:1000) yeast cells were spotted adjacently on YPD agar plates and incubated at 28 °C for approximately 72 h. The number of colonies was counted and calculated according to methods published in the literature [58].

### 3.4. Tolerance Assay

Yeast cells in the mid-log phase (10^6^ cells/mL) were reinoculated in fresh medium to make a suspension of yeast cells with a density of 10^5^ cells/mL, either with or without hyperoside (5 mg/L and 20 mg/L), and were incubated for 2 h at 28 °C/160 rpm. Then, the cells were subjected to oxidative stress (2 mmol/L H_2_O_2_, 10 mmol/L CCl_4_, or 3 mmol/L CdSO_4_) for 1 h. Cell viability was analysed by plating the appropriately diluted (1000×) cells in triplicate on solidified 2% YPD medium. The plates were incubated at 28 °C for 72 h, and then the colonies were counted. Survival expressed as a percentage was determined before and after oxidative stress exposure using cells treated with hyperoside and untreated control cells [59].

### 3.5. Cell Growth Assay

Yeast cells in mid-log phase (10^6^ cells/mL) were reinoculated in fresh medium and then treated with hyperoside for 2 h at 28 °C/160 rpm after treatment with oxidative stress for 1 h. The cells were plated in quadruplicate in 96-well plates and spotted on solid YPD media. The plates were incubated at 28 °C for 3 days before photography, and each assay was repeated at least three times [60].

### 3.6. Determination of Cell Membrane Lipid Peroxidation

Yeast cells (10^6^ cells/mL) exposed directly to oxidative stress for 1 h either pre-treated for 2 h with hyperoside or not treated were incubated at 28 °C /160 rpm. The cells (50 mg) were harvested by cooling on ice, centrifuged (5000 rpm/5 min/4 °C), and washed twice with distilled water. Cell pellets were resuspended in 0.5 mL of a 10% trichloroacetic acid solution (TCA) solution (*w*/*v*), and 1.5 g glass beads were added. Then, the cell walls were broken with cell breakers for 3 min at 5 degrees, and the supernatant was mixed with 0.1 mL of a 0.1 mol/L ethylenediaminetetraacetic acid (EDTA) solution and 0.6 mL of a 1% (*w*/*v*) thiobarbituric acid (TBA) solution in 0.05 mol/L NaOH. The reaction mixture was incubated in a boiling water bath for 15 min, and the mixture was cooled afterwards. The extracts were used to detect malondialdehyde (MDA) by measuring the absorbance at 532 nm [61,62].

### 3.7. Determination of Intracellular Oxidation

The intracellular oxidation level was assayed using the oxidant-sensitive probe 2′7′-dichlorofluorescein diacetate (H_2_DCF-DA) according to our previously established method [63]. Briefly, yeast cells with or without hyperoside were incubated for 2 h at 28 °C/160 rpm and then stressed with an oxidative agent for 1 h. The cells were incubated with the probe, harvested, washed, and resuspended in 0.5 mL water containing 1.5 g glass beads. Next, the cell walls were broken, and the supernatant solutions were centrifuged at 25,000× *g* for 5 min and diluted 6-fold with water before fluorescence was measured (an excitation wavelength of 504 nm and an emission wavelength of 524 nm).

### 3.8. Statistical Analyses

The statistical analyses were conducted using analysis of variance and Tukey’s test with the SPSS 12.0 package (SPSS, Inc., Chicago, IL, USA). The latter denoted homogeneity between the experimental groups at *p* < 0.05. To evaluate the levels of lipid peroxidation and intracellular oxidation, we compared homogeneity between stressed and non-stressed cells of each strain at *p* < 0.05. In all figures and tables, different letters indicate significantly different results.

## 4. Conclusions

Taken together, this study indicated that the dietary flavonoid hyperoside serves as a protector for *S. cerevisiae* in response to the stimulation of oxidative stress. More importantly, we identified that three antioxidant genes—CTT1, SOD1, and GSH—constructed a tight relationship between intercellular ROS and cellular viability regarding the antioxidant mechanisms of hyperoside through the yeast model. However, the three genes did not achieve a decrease in lipid peroxidation according to our results. It would be worthwhile to further investigate the mechanism to eventually better understand and develop dietary flavonoid products, including hyperoside.

## Figures and Tables

**Table 1:** Effect of 20 mg/L hyperoside on the level of ROS in yeast cells.

Stress	Treatment	WT	*sod1Δ*	*ctt1Δ*	*gsh1Δ*
CCl_4_	without hyperoside	4.77 ± 0.53 ^p^ a ^q^	3.35 ± 0.43b	2.49 ± 0.47c	2.41 ± 0.31c
	with hyperoside	1.62 ± 0.40d	1.69 ± 0.23d	2.36 ± 0.22c	1.45 ± 0.16d
H_2_O_2_	without hyperoside	14.23 ± 0.31a	6.99 ± 0.20b	5.84 ± 0.39c	8.87 ± 0.52b
	with hyperoside	4.39 ± 0.33c	1.87 ± 0.45d	5.86 ± 0.14c	1.24 ± 0.17d
CdSO_4_	without hyperoside	1.91 ± 0.23a	1.84 ± 0.56a	2.00 ± 0.15a	1.60 ± 0.30a
	with hyperoside	0.70 ± 0.57d	1.72 ± 0.24a	1.13 ± 0.17c	1.44 ± 0.29a

^p^ The results were expressed as a ratio between fluorescence of stressed, adapted or not with polyphenol, and non-stressed cells. The data represent the means ± SDs of at least three independent experiments. ^q^ Each stress was analyzed separately to determine statistical differences (different letters mean statistically different results at *p* < 0.05).

**Table 2:** Effect of 20 mg/L hyperoside on membrane lipid peroxidation in yeast cells.

Stress	Treatment	WT	*sod1Δ*	*ctt1Δ*	*gsh1Δ*
CCl_4_	without hyperoside	1.9 ± 0.3^p^a^q^	1.5 ± 0.2a	1.7 ± 0.2a	1.6 ± 0.3a
	with hyperoside	0.7 ± 0.2c	0.8 ± 0.3b	0.6 ± 0.3c	0.5 ± 0.2c
H_2_O_2_	without hyperoside	2.3 ± 0.3a	2.1 ± 0.2a	2.5 ± 0.2a	2.4 ± 0.3a
	with hyperoside	1.0 ± 0.2c	1.2 ± 0.3b	1.3 ± 0.1b	1.2 ± 0.2b
CdSO_4_	without hyperoside	1.5 ± 0.2a	1.2 ± 0.1a	1.3 ± 0.2a	1.4 ± 0.2a
	with hyperoside	1.1 ± 0.3b	0.6 ± 0.2c	0.8 ± 0.2c	0.7 ± 0.1c

^p^ The results were expressed as a ratio between lipid peroxidation levels of stressed, adapted or not with hyperoside, and non-stressed cells. The data represent the means ± SDs of at least three independent experiments. ^q^ Each stress was analyzed separately to determine statistical differences (different letters mean statistically different results at *p* < 0.05).

## Supplementary Materials

The following are available online at https://www.mdpi.com/1420-3049/24/4/788/s1, Figure S1: The 1H-NMR spectra (DMSO, 400 MHz) of hyperoside, FigureS2: The 13C-NMR spectra (DMSO, 100 MHz) of hyperoside, Figure S3: The HPLC spectra of hyperoside, Table S1: The analytical result of hyperoside.

## Abbreviations

SODs	superoxide dismutases
GPXs	glutathione peroxidases
CTT1	cytosolic catalaseT1
Prxs	peroxiredoxins
GSH	glutathione
ROS	reactive oxygen species
*S.cerevisiae*	saccharomyces cerevisiae
GTT1	glutathione transferase1
GTT2	glutathione transferase2
YPD	yeast extract peptone dextrose
TCA	trichloroacetic acid
TBA	thiobarbituric acid
MDA	malondialdehyde
H_2_DCF-DA	2′7′-dichlorofluorescein diacetate
WT	wild type
